# Hand Hygiene Practices during the COVID-19 Pandemic in Northern Italy: Assessment of Compliance Rates Measured by Direct Observation and Alcohol-Based Handrub Usage

**DOI:** 10.3390/antibiotics11111510

**Published:** 2022-10-29

**Authors:** Costanza Vicentini, Giulia Libero, Valerio Bordino, Carla Maria Zotti

**Affiliations:** Department of Public Health and Paediatrics, University of Turin, Via Santena 5 bis, 10126 Turin, Italy

**Keywords:** hand hygiene, COVID-19, Italy, infection control, healthcare-associated infections

## Abstract

Hand hygiene (HH) is among the most effective measures for reducing the transmission of healthcare-associated infections and SARS-CoV-2. We aimed to assess HH practices among healthcare workers (HCWs) of three hub hospitals in Northern Italy during the COVID-19 pandemic, by assessing HH compliance measured by direct observation and alcohol-based handrub usage. An observational study was conducted over a period of three months, between February and April 2021. HH compliance audits were conducted using the WHO My 5 Moments for HH approach. Multivariable logistic regression was used to evaluate independent predictors of HH compliance: ward type, HCW category and HH indication. Spearman correlation was used to investigate the relationship between HH compliance and alcohol-based handrub consumption. In total, 2880 HH opportunities were observed, with an overall compliance of 68%. Significant differences were found in compliance rates across ward types, HCW categories and HH indications. The mean alcohol-based handrub usage among included wards was 41.63 mL/PD. No correlation was identified between compliance rates and alcohol-based handrub consumption (ρ 0.023, p 0.943). This study provided a snapshot of HH practices in a pandemic context, which could be useful as a reference for future studies.

## 1. Introduction

Healthcare associated infections (HAIs), along with antibiotic resistance, are a leading risk for patient safety, increasing morbidity, mortality and healthcare costs [1,2], and are associated with a significant burden of disease [3,4]. According to recent estimates, more than 2.6 million cases of HAIs occur in the European Union each year [4]. 

Hand hygiene (HH) is considered to be among the most effective and cost-effective measures for the reduction of HAIs [2]. In order to promote HH, in 2005 the WHO initiated the global ‘Clean care is safer care’ HH campaign [1]. In Italy, a national multimodal HH campaign based on the tools provided by the WHO was launched in November 2006. In the region of Piedmont, in Northern Italy, HH practices are routinely monitored as part of the mandatory performance indicator system for HAI prevention. 

Northern Italy was among the European regions most heavily impacted by the recent COVID-19 pandemic [5]. As transmission modes of SARS-CoV-2 include contact and droplet transmission, HH is a crucial preventive measure [6]. Recent data suggest the pandemic has led to improvements in HH compliance in healthcare settings [7,8,9], although the impact of COVID-19 on infection prevention and control (IPC) practices remains to be determined [10,11]. The aim of this study was to assess HH practices among healthcare workers (HCWs) of three hub hospitals in Northern Italy during the later stage of the COVID-19 pandemic, by assessing HH compliance measured by direct observation (using the WHO My 5 Moments for HH approach [12]) and alcohol-based handrub usage. As IPC resources were significantly diverted to outbreak response during the pandemic, and direct observation of HH is time and resource-consuming, we aimed to evaluate whether handrub consumption could be used as a surrogate marker for HH.

## 2. Results

In total, 2880 HH opportunities were observed, with an overall compliance of 68%. In 90% of complied opportunities, alcohol-based handrub was used, and water and soap in the remaining 10%. Detailed descriptive statistics are presented in Table 1. The highest compliance rate was observed in intensive care units (ICUs), the lowest in medical wards. The mean compliance rate in non-ICUs was over ten percentage points below the compliance rate in ICUs (64.7% vs. 76%). Considering HCW categories, the highest compliance rates were observed among nurses, followed by ancillary staff and physicians. An important variability was observed among HCWs according to ward type. An almost progressive increase in compliance was observed with each of the five moments for HH, with the lowest compliance for moment 1 (before patient contact) and reaching the highest level for moment 5 (after contact with patient surroundings).

Results of the multivariable logistic analysis are presented in Table 2. A significant association was found between ward type and HH compliance, with more than tripled odds of compliance in ICUs, and more than doubled odds of compliance in day hospitals and surgical wards compared to medical wards. Doctors were associated with reduced odds of compliance of 32% compared to nurses (*p* < 0.01). Considering the first moment for HH as reference, all of the following moments were associated with higher odds of compliance, although a significant result was found for moments 4 and 5, after patient contact and after contact with patient surroundings (OR 1.41, 95% CI 1.14–1.75, *p* = 0.002, and OR 2.38, 95% CI 1.93–2.93, *p* < 0.01, respectively).

**Figure 1:**
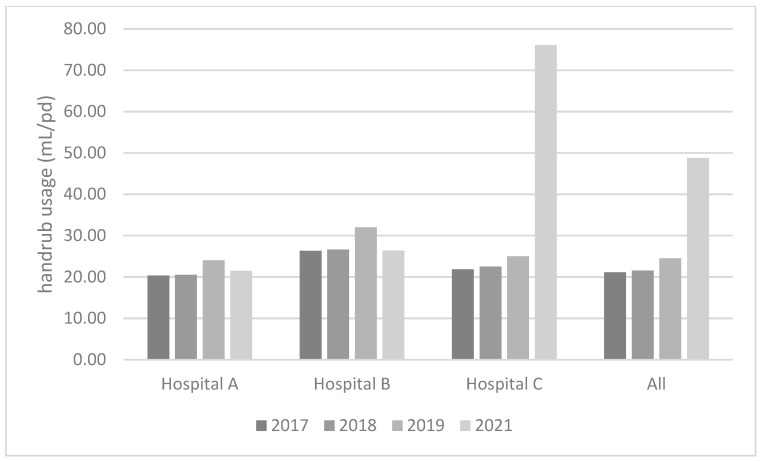
Mean alcohol-based handrub usage in included hospitals, February-April 2017–2019 vs. 2021.

The mean alcohol-based handrub usage among included wards was 41.63 mL/Patient-Days (PD) (range 4.48–158.42). As shown in Table 3, the highest consumption of alcohol-based handrub according to ward type was found in ICUs (mean 73.93, range 30.15–158.42) and the lowest in day hospitals (mean 7.86, range 4.48–14.38). No correlation was identified between compliance rates and alcohol-based handrub consumption, both considering all data (ρ 0.023, p 0.943) and excluding outliers (ρ 0.212, p 0.556). Figure 1 shows mean alcohol-based handrub usage per hospital, during the years 2017–2019 vs. 2021. A slightly increasing trend was identified for years 2017–2019 among all hospitals. Comparing 2021 to the previous years, an important increase was observed in handrub usage for Hospital C, but usage slightly decreased compared to 2019 in Hospitals A and B. While Hospital A and B are specialised hospitals in gynaecological and paediatric care, Hospital C is a hub general hospital. During COVID-19 pandemic in 2021 there were different wards dedicated to the care of patients with COVID-19 in Hospital C. Thus, the increased handrub usage in Hospital C in 2021 might be due to the high prevalence of COVID-19 patients and to the high focus on good practices during patient care. 

## 3. Discussion

Monitoring HH practices produces important data that can be used to provide feedback to HCWs, to identify areas for improvement, and to evaluate the effectiveness of targeted interventions [13]. The COVID-19 pandemic has put IPC efforts under severe stress [11]. A survey conducted among HCWs of two hospitals in Wuhan in the early stage of the outbreak found an improvement of self-reported IPC behavior, particularly in regard to HH [14]. Recent studies have found improved HH compliance rates during the pandemic [7,8,9], which might have been driven by increased public and political attention to communicable diseases, increased awareness of the importance of HH as a preventive measure, and heightened personal risk perception among HCWs, as well as factors related to work conditions [8,9,10]. In the three hospitals examined in this study, focus on HH was also considerably enhanced at all levels and several HH promoting initiatives were undertaken, such as educational interventions, auditing, and displaying of posters. We aimed to provide a snapshot of HH adherence rates in this context, which could be useful as a reference for future studies. 

In our study, an overall compliance of 68% was found, which was in line with findings of Moore et al. during the pandemic, notwithstanding methodological differences [8]. In the studies by Makhni et al. and Wong et al., peaks of 100% were achieved during the pandemic, although these results were not sustained over time across all wards [7,9]. For comparison, a systematic review of HH-related clinical trials published in 2016 estimated a mean baseline compliance rate of 34.1% [15], and a nationwide study of German hospitals reported a median consumption of 35.9 mL/PD in 2015 [16]. The mean alcohol-based handrub usage among all hospitals in 2021 was 41.63 mL/PD, with an important increase compared to years 2017–2019. However, it must be noted that an increasing trend was identified for years 2017–2019, whereas there was a slight decrease in 2021 in two out of the three included hospitals. It will be interesting to investigate how the trend evolves in the future.

Consistent with previous studies, we found significant differences in compliance rates across ward types, HCW categories and HH indications. In our study, compliance was higher in ICUs compared to non-ICUs. This finding is notable, as activity levels and workloads are higher in these settings, usually leading to lower or similar compliance in ICUs compared to non-ICUs [17]. ICUs also had the highest handrub usage in our study, in line with previous reports [17,18], which could be explained by a greater number of HH indications in these settings. Our results support the widely reported observation of higher compliance rates among nurses compared to physicians [19,20], Concerning HH indication, in our study, the moment associated with the highest compliance was moment 5 (after contact with patient surroundings). Previous studies have found higher compliance with HH after patient contact compared to before patient contact in general, suggesting self-protection rather than patient safety is a main driver for HH compliance among HCWs, although the last moment is usually overlooked [19,21,22]. The increased perception of risk due to COVID-19, and in particular the heightened attention to the role of the environment in SARS-CoV-2 transmission, could contribute to explain the increased compliance we found for moment 5. Moment 1, before patient contact, had the lowest compliance in our study, reinforcing the concern that the focus of HCWs during the pandemic remained self-protection rather than preventing cross-transmission between patients [8,10].

Direct observation is considered the gold standard for HH monitoring [1], but it is resource consuming and subject to considerable bias and limitations, as it requires observers to be present during all stages of patient care [23,24,25]. During the pandemic, the diversion of personnel to outbreak management and shortages of HCWs with appropriate IPC training have affected healthcare facilities’ ability to gather IPC data, including process measures such as HH compliance [10,11]. Further, changes in workflow and isolation precautions have limited the feasibility of performing direct observation sessions in high-risk settings. Therefore, we investigated whether alcohol-based handrub usage could be used as surrogate indicator for HH compliance in this context. This strategy has been evaluated in other settings, as data on product usage are relatively simple to obtain and no additional staff or other resources are required [25]. Further, this metric is not subject to the Hawthorne effect [15,20].

In our study, no correlation between product usage and compliance was identified, in line with previous reports [18,24]. Marra et al. suggested that product usage could be more representative of the true rate of hand hygiene adherence compared to direct observation, due to the inherent limitations of this method [24]. Magnus et al. also found no correlation between the two metrics but concluded that both are necessary to provide a more accurate assessment of HH performance [18].

Direct observation provides important information that cannot be obtained by solely measuring product usage, as it allows to identify particular HCW groups and HH behaviors for which targeted interventions may be required [22]. Observation sessions provide a platform for HH education, and the My 5 Moments for HH approach provides an evidence-based conceptual framework for interventions [11]. Further, alcohol-based handrub usage estimates can be inaccurate, due to the removal or redistribution of product, or to its use by patients and visitors [15,17]. Finally, direct observation not only allows to measure HH adherence, but it also has an enhancing effect on compliance rates [23,24]. 

This study had several limitations that must be considered when interpreting results. Limitations of both metrics, direct observation and alcohol-based handrub usage, have been discussed. Further, there were some limitations due to study design and methodology for data collection: (1) observation sessions were performed predominantly during morning shifts and therefore do not represent a complete picture of 24h ward activity, although these shifts are usually the most intense in terms of workload and activity levels; (2) using the My 5 Moments for HH approach does not provide information on the quality of performed HH; (3) we did not evaluate the impact of other unmeasured factors that could be associated with HH compliance. Finally, as it was not the purpose of this study to evaluate the impact of HH practices on HAI rates, we make no assumption on the validity of the metrics considered in this study as indicators for pathogen transmission or infection risk. Further research is required to investigate these aspects.

## 4. Materials and Methods

### 4.1. Study Design and Setting

This observational study was conducted over a period of three months, between February and April 2021, in three large, tertiary-care, academic centers of Turin, Italy. The study was set in twelve wards: three ICUs, three surgical wards, three-day hospitals, and three medical wards. As data concerning HH are routinely collected for the regional performance indicator system, with the objective of improving healthcare quality and patient safety, no institutional review board approval was required for this study.

### 4.2. HH Compliance – Definitions and Data Collection

HH compliance audits were conducted in the twelve included wards using the WHO My 5 Moments for HH approach [12]. Observation sessions in each ward lasted one hour per day and included a minimum of 200 observations, with two of the study authors, IC and GL, serving as overt observers. The observers were trained prior to the study and were flanked by infection control nurses experienced in HH compliance monitoring via direct observation. Doctors, nurses, ancillary staff, and any other HCWs providing care in the included wards were observed.

HH compliance was defined as the action of washing hands or using alcohol-based handrub and was recorded for each HH opportunity. The WHO My 5 Moments for HH approach was used to identify indications for HH: before patient contact, before aseptic procedure, after body fluid contact, after patient contact, after contact with patient surroundings. Compliance (coded as yes or no) and type of performed HH (with alcohol-based handrub, with water and soap, or not performed) for each opportunity were recorded in real time using the app SpeedyAudit (HandyMetrics Corporation, Toronto, ON, Canada). The type of unit in which the observation session took place and the category of observed HCW were noted, but all data were de-identified. Observers informed observed participants that they were monitoring infection control practices.

### 4.3. Alcohol-Based Handrub Usage

The total volume of alcohol-based handrub acquired by each included ward in the considered three months was obtained from the hospital pharmacy. Patient-days (PDs) were calculated for each ward as the total length of hospital stay in days of all patients admitted during the study period, or as the total number of accesses (considered as one patient-day per access) in the case of day hospitals. Alcohol-based handrub usage for each ward was expressed in milliliters per PD.

### 4.4. Statistical Analysis

HH compliance was calculated as a percentage (number of complied HH opportunities/total number of HH opportunities). Descriptive statistics were used to assess HH compliance rates according to ward type, HCW category and HH indication. Multivariable logistic regression was used to evaluate independent predictors of HH compliance. Relevant variables were inserted in the model with enter method. The association between compliance and explanatory variables was measured by odds ratios (ORs) and corresponding 95% confidence intervals (CIs). Spearman correlation was used to investigate the relationship between HH compliance and alcohol-based handrub consumption. A two-tailed p value of 0.05 was considered significant. All analyses were conducted using SPSS Version 27.0 (SPSS Inc., Armonk, NY, USA).

## 5. Conclusions

In conclusion, HH has received much attention in the context of the COVID-19 pandemic as an important tool for both HCW and patient safety. Our study adds to the cur-rent literature by investigating a variety of settings in a pandemic context, providing data that could be useful for benchmarking purposes in future studies evaluating the long-term impact of COVID-19 on IPC practices. Future research should be dedicated to evaluating the impact of compliance with HH among medical personnel on reducing the risk of HAIs and SARS-CoV-2 infection.

Further, this study identified a number of target areas for quality improvement interventions. Authors have recommended that health systems should consider creative methods to support IPC practices during the pandemic [11], although this study does not support the validity of product usage as a surrogate marker for HH compliance. Beyond COVID-19, using both indicators should be considered, as they provide complementing information [18,24].

## Figures and Tables

**Table 1:** Number of hand hygiene (HH) opportunities and compliance rate according to ward type, health-care worker (HCW) category and HH indication in three hub hospitals in Northern Italy, February–April 2021.

	N of Observations	Compliance Rate
*Ward type*		
Medical unit	620	49%
Day Hospital	857	75%
Surgical unit	712	70%
Intensive care unit	692	76%
*HCW category*		
Nurse	1869	72%
Physician	596	59%
Ancillary staff	384	66%
Others	32	72%
*Indication for HH*		
Moment 1	780	58%
Moment 2	193	67%
Moment 3	126	69%
Moment 4	718	67%
Moment 5	1063	77%

**Table 2:** Multivariable analysis–predictors of hand hygiene (HH) compliance.

	OR (95% CI)	*p*
*Ward type*		
Medical unit	REF.	
Day Hospital	2.62 (2.07–3.32)	<0.01
Surgical unit	2.15 (1.7–2.72)	<0.01
Intensive care unit	3.32 (2.6–4.24)	<0.01
*HCW category*		
Nurse	REF.	
Physician	0.68 (0.55–0.84)	<0.01
Ancillary staff	0.88 (0.68–1.14)	0.359
Others	1.01 (0.44–2.29)	0.974
*Indication for HH*		
Moment 1	REF.	
Moment 2	1.38 (0.97–1.95)	0.068
Moment 3	1.46 (0.96–2.22)	0.070
Moment 4	1.41 (1.13–1.75)	0.002
Moment 5	2.37 (1.92–2.93)	<0.01

**Table 3:** Alcohol-based handrub usage according to ward type, February–April 2021.

	Mean Handrub Consumption (Range), mL/Patient-Day
Medical wards	32.1 (30.84–33.15)
Day Hospitals	7.86 (4.48–14.38)
Surgical wards	52.64 (12.82–125.64)
Intensive care units	73.93 (30.15–158.42)

## Data Availability

Data will be made available upon reasonable request.
